# The Construction of a New Bladder

**Published:** 1889-03

**Authors:** 


THE CONSTRUCTION OF A NEW BLADDER from a part of the intestinal canal, is the latest achievement of operative surgery, although so far only successfully executed by G. Tizzoni and A. Foggi on a female dog.

The operation was performed *en deux temps*. First, laparatomy and exsection of a piece of ileum, which was antisepticised and closed on both ends, while the continuity of the intestine was restored in the usual manner. One month later, the second operation took place, the ureters were isolated from the bladder, implanted into the piece of gut, the bladder extirpated and the urethra attached to an incision into the isolated loop of intestine, which had contracted considerably. Incontinence for fifteen days; later continence followed, so that the dog voided the urine voluntarily about every hour. Evidently a loop of seven cm. was not long enough. For further detail, see original article in the *Centralblatt für Chirurgie*, No. 50. 1888. M. H.

